# Evaluation of health-promoting self-care behaviors in hypertensive patients with concomitant chronic kidney disease in primary care

**DOI:** 10.1017/S1463423622000299

**Published:** 2022-08-24

**Authors:** Oleksii Korzh, Anna Titkova, Yana Fylenko, Yelizaveta Lavrova

**Affiliations:** Department of General Practice-Family Medicine, Kharkiv Medical Academy of Postgraduate Education, Kharkiv, Ukraine

**Keywords:** chronic kidney disease, family physician, health behavior, hypertension, primary care, quality of life, self-efficacy

## Abstract

**Aim:**

This study was to investigate the relationships among health behaviors and quality of life (QOL) and to test a proposed model among people with hypertension and concomitant chronic kidney disease (CKD) in primary care. In addition, the mediation effect of modifiable risk factors between self-care health behaviors and QOL was examined.

**Methods:**

This study was prospective, conducted in the centers of primary medical care in the period from January 2018 to January 2020. In total, 170 patients diagnosed with hypertension and CKD at least 12 months previously were included in this study. The following parameters were measured: self-efficacy, self-care health behaviors with the subscales of health responsibility, exercise, consumption of a healthy diet, stress management, and smoking cessation; modifiable risk score; and QOL (assessed using the 36-item Short-Form Health Survey instrument).

**Results:**

Self-efficacy had a significantly positive direct effect on self-care health behaviors, with a standardized regression coefficient of 0.87 (*P* = 0.007), a negative indirect effect on risk factors, with a standardized regression coefficient of 0.11 (*P* = 0.006), and a positive indirect effect on QOL, with a standardized regression coefficient of 0.62 (*P* = 0.008). Self-care health behaviors had a significantly positive direct effect on QOL, with a standardized regression coefficient of 0.72 (*P* = 0.012); there was also an indirect effect of 0.053 (*P* = 0.004). The direct effect of risk factors on QOL was significant, with a standardized regression coefficient of 0.44 (*P* = 0.018). The direct effect of self-care health behaviors on QOL was 0.77 (*P* = 0.008), which has been reduced to 0.72 (*P* = 0.012). The reduced effect of 0.05 was significant (*P* = 0.004), confirming the mediating role of modified risk factors.

**Conclusions:**

This study indicates health-promoting behaviors in hypertensive patients with CKD have a potential impact on their QOL in primary care. Primary care physicians should focus on motivation strategies to encourage individuals to perform self-care health-promoting behaviors associated with the improved QOL, in order to achieve better outcomes in risk factor management.

## Introduction

Hypertension is one of the main causes of chronic kidney disease (CKD), which affects almost one in seven people. The observed increase in the incidence of CKD is partly associated with an increase in the prevalence of diabetes, obesity, and hypertension (Hanratty *et al*., 2011; Walther *et al*., 2019). Hypertension, the second leading cause of end-stage renal disease (ESRD), is a major risk factor for the progression of CKD and the most common concomitant pathology in CKD (Hanratty *et al*., 2011). Identification of risk factors for the progression of CKD in patients with hypertension may help targeted therapy to slow down or prevent a decrease in kidney function.

Many risk factors for hypertension are lifestyle related. Therefore, the focus of treatment for chronic diseases should be changed from medical treatment to improving the lifestyle of people during treatment. To achieve this goal in patients with chronic diseases, health-promoting measures are recommended as a strategy to control the cost of medical care and improve the quality of life (QOL) (Warren-Findlow and Seymour, 2011; Han *et al*., 2014). Health-promoting behaviors, as one of the key determinants of health (Warren-Findlow and Seymour, 2011), are the behaviors or actions that people perform in an effort to improve their health (Han *et al*., 2014). Health-promoting behavior is behavior in which a person receives adequate nutrition, performs regular physical exercises, avoids malicious behavior and drugs, protects against accidents, timely diagnoses symptoms in the physical aspect, controls emotions and thoughts, adapts to situations, relieves stress, and corrects interpersonal and social relations (Warren-Findlow and Seymour, 2011).

Current CKD recommendations are not age related and widely recommend that patients actively participate in health-related behaviors to manage their disease and mitigate its effects. These behaviors include monitoring blood pressure, quitting smoking, maintaining a healthy weight, eating healthy, and participating in physical activity (Stevens and Levin, 2013; Montoya *et al*., 2016). The effectiveness of these health behavioral recommendations for CKD is relatively unknown, as they were obtained from studies in the general population (Vassalotti and Kaufman, 2013). In addition, to date, there is limited evidence of behavioral practices regarding the health of adults with CKD (Jha *et al*., 2013). In particular, even though older people are likely to experience unique challenges to health problems, such as higher levels of comorbid conditions, weakness, and reduced functional status, little attention is given to whether or not health behavioral acceptance is different for older compared to young. Such information is important for understanding care gaps and developing age-appropriate recommendations and interventions to help all patients with CKD.

The QOL is of paramount importance in connection with the consideration of several aspects, such as physiological aspects and performance, as indicators related to health (Habibi *et al*., 2008). Evaluation of QOL in patients with hypertension with CKD is very important (Borges *et al*., 2017). The results of some studies showed that most patients with hypertension had a low level of QOL (Ahn *et al*., 2016; Kitaoka *et al*., 2016). To improve QOL in people with hypertension, attention should be paid to factors affecting QOL, including health-promoting behaviors, as a way to identify problems associated with lifestyle changes and improving QOL (Borges *et al*., 2017). Because of the importance of this issue, the aims of this study were to investigate the relationships among health behaviors and QOL and to test a proposed model among people with hypertension and concomitant CKD in primary care. In addition, the mediation effect of modifiable risk factors between self-care health behaviors and QOL was examined.

## Materials and methods

This study was prospective, conducted in the centers of primary medical care in Kharkiv (clinical bases of the Kharkov Medical Academy of Postgraduate Education) in the period from January 2018 to January 2020.

In total, 170 patients were included in this study. Potential participants were recruited using the following inclusion criteria: (a) diagnosed with hypertension and CKD at least 12 months previously, (b) were able to communicate and understand the questionnaire, and (c) agreed to participate in the survey with written consent.

The study was conducted in accordance with international standards of bioethics (Council of the European Convention on Human Rights and Biomedicine) and the recommendations of the Committee on Bioethics of the Ministry of Health of Ukraine. All patients signed an informed consent to participate in the study. This study was approved by the Ethics Commission of the Kharkov Medical Academy of Postgraduate Education of the Ministry of Health of Ukraine (Kharkiv, UA).

### Self-care health behaviors

The cardiac health behavior scale was developed by Oh *et al.* (2013) and modified by the researchers. The modified CHBS covered the dimensions of (1) physical activity, (2) stress management, (3) diet management, (4) smoking cessation, (5) blood pressure control, and (6) taking medications. Three items were included in this study as they measured taking medications. Three items were excluded in this study as they measured heart responsibility, which was not the focus of this study. The questionnaire consisted of 25 items; it used a 4-point response (1 = never, 2 = sometimes, 3 = often, 4 = routinely), detailed as follows.

- Never: A person never performed the behavior
- Sometimes: A person performed the behavior 1–3 days per week
- Often: A person performed the behavior 4–6 days per week
- Routinely: A person performed the behavior every day.

The scores were then computed by summing up scores from the items. Possible scores ranged from 25 to 100. A higher score represented a better performance of cardiovascular health behavior. Furthermore, the MCHBS scores were divided into three groups: scores 25–50 indicated the low performance of cardiovascular health behavior; scores 51–75 indicated the moderate performance of cardiovascular health behavior, and scores 76–100 indicated the high performance of cardiovascular health behavior.

### Self-efficacy

Self-efficacy was measured using a subscale of the Motivation Scale for Health Behavior. The perceived self-efficacy scale comprised six items that were scored on a 10-point numerical rating scale, from 1 (not confident at all) to 10 (very confident). Higher scores in the perceived self-efficacy scale represent a more confident state for performing health behaviors.

### Modifiable cardiovascular risk factors

The summed scores for modifiable cardiovascular risk factors were calculated based on the cardiac risk factor profile and the sum of weighted scores for modifiable risk factors such as cholesterol, glucose, systolic blood pressure, obesity (BMI and waist/hip ratio), smoking habit, stress, and exercise. The profile was modified according to a gender-specific multivariable risk factor algorithm (D’Agostino *et al*., 2008). Physiological variables including blood pressure and anthropometric parameters were measured by an exercise physiologist, and fasting blood for glucose and lipid profile was sampled and processed at a research laboratory.

### QOL

QOL was measured using version 2.0 of the Short Form Health Survey (Ware *et al*., 2002). Our version of the form was consisted of the weighted summed scores of physical and mental components with norm-based scoring algorithms of standard form (4-week recall). A higher score indicates a better QOL in the corresponding dimension.

### Data analysis

The collected data were analyzed to describe the demographic characteristics, the main study variables, and the relationships among the study variables. The following assumptions were examined before *Structural Equation Modeling* (SEM): the absence of influential cases, multivariate normality, linearity, and homoscedasticity. *SEM* was conducted using AMOS 19.0 software (IBM Corp.; Armonk, NY, USA) to examine the direct and indirect effects of self-efficacy, self-care health behaviors, and modifiable risk factors on the dependent variable (QOL). None of the assumptions were violated. All *P* values were calculated using the bootstrapping method in the AMOS software, which was also used to test the mediating effect of modifiable risk factors between self-care health behaviors and QOL. The procedures outlined by Baron and Kenney (1986) were used to test these mediation hypotheses. Significance testing of the mediation effect was achieved using the bootstrapping method in AMOS to test indirect effects.

## Results

### Sociodemographic characteristics of participants

The participants were aged 64.22 years (± 5.29 years; range: 37–78 years). Approximately half of the participants (54.7%) were female, and most (79.4%) were married and living with their partner. The educational status of the sample varied, and the highest levels of education reached being middle school, high school, and university (bachelor or higher) in 34.1%, 32.4%, and 33.5% of the cohort, respectively. More of the participants perceived their economic status as being moderate or high (62.9%) (Table 1).

**Table 1.** Characteristics of patient with hypertension and chronic kidney disease (*n* = 170)

Characteristics	Categories	Frequency (%)
Gender	Male	77 (45.3)
	Female	93 (54.7)
Marital status	Married	135 (79.4)
	Other	35 (20.6)
Education	Middle school	58 (34.1)
	High school	55 (32.4)
	College or higher	57 (33.5)
Economic status	Moderate or higher	107 (62.9)
	Low	63 (37.1)
Smoking	Never smoked	87 (51.2)
	Quit smoking	66 (38.8)
	Currently smoking	17 (10)
Diabetes	Yes	42 (24.7)
	No	128 (75.3)
Dyslipidemia	Yes	82 (48.2)
	No	88 (51.8)
Perceived health status	Very poor	34 (20)
	Poor	47 (27.6)
	Moderate	49 (28.8)
	Good	29 (17.1)
	Excellent	11 (6.5)

Regarding the health-related characteristics of the participants, about half (51.2%) had never smoked, and only 10.0% were still smoking. In addition, 24.7% of the sample reported having diabetes and 48.2% reported having dyslipidemia. Their perceived health status varied from very poor (20%), poor (27.6%), moderate (28.8%), good (17.1%), to excellent (6.5%).

### Levels and relationships among main study variables

The means, standard deviations, and a correlation matrix with zero-order correlations among the study variables are presented in Table 2. On the 10-point Likert scale, participants reported a moderate level of self-efficacy (7.17 ± 1.85), and among the self-care health behaviors, scores on all of the 4-point subscales were at the moderate level, with smoking cessation scoring the highest (3.79 ± 0.68). The score for modifiable risk factors was 10.39 ± 3.24, indicating a moderate level of risk. Scores on the dependent variable of QOL were also at a moderate level, at 45.16 ± 8.23 for physical component summary (PCS), and 42.64 ± 8.12 for mental component summary (MCS).

**Table 2.** Levels and interrelationships among study variables (*n* = 170)

No.	Variables	Mean± SD	*r*(*p*)								
			1	2	3	4	5	6	7	8	9
1	Self-efficacy	7.17 ± 1.83	1								
2	Health responsibility	2.99 ± 0.81	0.57(<0.001)	1							
3	Exercise	2.88 ± 0.92	0.71(<0.001)	0.44(<0.001)	1	1					
4	Healthy diet	3.45 ± 0.65	0.54(<0.001)	0.58(<0.001)	0.49(<0.001)	0.46(<0.001)					
5	Stress management	2.81 ± 0.72	0.41(<0.001)	0.42(<0.001)	0.28(0.003)	0.31(0.001)	1				
6	Smoking seccation	3.79 ± 0.68	0.31(0.002)	0.16(0.122)	0.11(0.475)	0.09(0.481)	0.22(0.039)	1			
7	Modifiable risk factors	10.39 ± 3.24	−0.16(0.106)	0.11 (0.316)	0.21(0.062)	0.18(0.096)	0.06(0.688)	−0.011(0.402)	1		
8	QOL PCS	45.16 ± 8.23	0.27(0.008)	0.13(0.892)	0.31(0.001)	0.22(0.061)	0.07(0592)	0.06(0.614)	−0.22(0.058)	1	
9	QOL MCS	42.64 ± 8.12	0.24(0.022)	0.14(0.312)	0.18(0.096)		0.31(0.001)	−0.04(0.720)	−0.24(0.455)	0.14(0.165)	1

QOL MCS = quality of life mental component summary; QOL PCS = quality of life physical component summary.

Except for modifiable risk factors, all other study variables were significantly correlated with each other. Self-efficacy was positively related to all subscales of self-care health behaviors and QOL. Each self-care health behavior was partially associated with the QOL component (Table 2).

### Direct and indirect effects of study variables on QOL in hypertensive patients with CKD

Self-efficacy, self-care health behaviors indicated by the five subscales, modifiable risk factors, and QOL indicated by PCS and MCS were submitted to SEM analysis using AMOS. The resulting model yielded a significant χ^2^ value of 59.64 (df = 26, *P* < 0.001), suggesting an inadequate fit between the sample and the implied covariance matrices. However, χ^2^ statistics are very sensitive to large sample sizes and data distribution, and relative χ^2^ (the χ^2^ index divided by the degree of freedom) was also used to evaluate model fit because this index might be less sensitive to sample size. In this study, the relative χ^2^ was 2.36, suggesting this model is regarded as acceptable since the criterion for acceptance was less than 3.

Self-efficacy accounted for 76.0% of the variance associated with self-care health behaviors, while self-care health behaviors and modifiable risk factors together accounted for 72.0% of the variance in QOL. Standardized regression coefficients (both direct and indirect) for the structural model analysis are listed in Table 3. Self-efficacy had a significantly positive direct effect on self-care health behaviors, with a standardized regression coefficient of 0.87 (*P* = 0.007), a negative indirect effect on risk factors, with a standardized regression coefficient of 0.11 (*P* = 0.006), and a positive indirect effect on QOL, with a standardized regression coefficient of 0.62 (*P* = 0.008). Self-care health behaviors had a significantly positive direct effect on QOL, with a standardized regression coefficient of 0.72 (*P* = 0.012); there was also an indirect effect of 0.053 (*P* = 0.004). The direct effect of risk factors on QOL was significant, with a standardized regression coefficient of 0.44 (*P* = 0.018).

**Table 3.** Direct and indirect effects of independent variables on dependent variables (*n* = 170)

Independent variables	Dependent variables^a^				
	Self-care health behaviors	Modifiable risk factors		Quality of life	
	Direct effect	Direct effect	Indirect effect	Direct effect	Indirect effect
Self-efficacy	0.87 (0.007)		−0.11 (0.006)		0.62 (0.008)
Self-care health behaviors		−0.14 (0.008)		0.72 (0.012)	0.053 (0.005)
Modifiable risk factors				−0.44 (0.018)	

a Data are standardized regression weights with *P* values as calculated using bootstrap methods.

### Effects of modifiable risk factors mediating between self-care health behaviors and QOL

One of the study hypotheses required testing of the effects of risk factors in mediating self-care health behaviors and QOL. This was achieved using the procedures outlined by Baron and Kenney (1986), according to whom the following three conditions must be met for a variable to be considered as a mediator: (a) the predictor variable must be significantly associated with the hypothesized mediator, (b) the mediator must be significantly associated with the dependent variable after controlling for the predictor, and (c) the impact of the predictor variable on the dependent variable becomes nonsignificant (supporting the presence of a complete mediating effect) or significantly reduced (supporting the presence of a partial mediating effect) when the mediator is controlled.

The first condition was satisfied; the variable of self-care health behaviors was significantly associated with the mediator (modifiable risk factors; *β* = −0.14, *P* = 0.008). The second condition of mediation required that the mediator (risk factors) be significantly associated with the dependent variable of QOL after controlling for self-care health behaviors. The second condition was also satisfied. The variable of modifiable risk factors was significantly associated with QOL after controlling for self-care health behaviors (*β* = −0.44, *P* = 0.018). The third condition of mediation required that the impact of the predictor variable (self-care health behaviors) on the dependent variable (QOL) was nonsignificant or reduced significantly after controlling for the mediator of risk factors; this condition was also satisfied. The direct effect of self-care health behaviors on QOL was 0.77 (*P* = 0.008), which has been reduced to 0.72 (*P* = 0.012). The reduced effect of 0.05 was significant (*P* = 0.004), confirming the mediating role of modified risk factors. These results indicated that all three conditions for mediation were satisfied, indicating that modifiable risk factors mediate the relationship between self-care health behaviors and QOL. Since the impact of self-care health behaviors on QOL was reduced significantly, it can be concluded that risk factors partially mediate the effect of self-care health behaviors on QOL.

## Discussion

This study aimed to examine the association between self-care behavior and QOL in hypertensive patients with concomitant CKD in primary care. The results showed that modifiable risk factors can be more effectively managed by the individuals performing the self-care health behaviors.

In most previous studies, a significant relationship has been reported between self-care and blood pressure control in which a self-care relationship was observed on systolic and diastolic blood pressure reduction (intervention studies) (Cappuccio *et al*., 2004; Carter *et al*., 2009; Padfield, 2010; Victor *et al*., 2011; Korzh and Krasnokutskiy, 2016). There are limited studies about the relationship of self-care with QOL, which is an principal outcome of hypertension control; however, the impact of hypertension on QOL has been reported by Cappuccio *et al*., so that these patients have a lower QOL, mostly due to the treatment of hypertension rather than the disease itself (Cappuccio *et al*., 2004).

Self-care is defined as any action taken to maintain one’s health and prevent disease, including health, nutrition, and lifestyle, environmental conditions, income and socio-economic status, and self-treatment (Han *et al*., 2014). Self-care in patients with high blood pressure has been announced as a key step in reducing hypertension pandemic (Warren-Findlow *et al*., 2013; Korzh *et al*., 2019). Self-care behavior is considered a major determinant of blood pressure control in some studies. In other studies, behaviors such as proper use of medications, exercise, proper nutrition, and weight control are further examples of self-care. However, factors other than age, sex, marital status, employment, duration of hypertension, knowledge and beliefs about it, and self-efficacy related to self-care and hypertension have been emphasized (Korzh *et al*., 2019). Some studies have examined the impact of chronic diseases on QOL. It has been reported that people with hypertension have lower QOL compared to those with normal blood pressure (Warren-Findlow *et al*., 2013).

Self-efficacy, which plays an important role in reducing the progression of CKD, has been included in various approaches for use in people with a diagnosis of CKD at all stages (Tangri *et al*., 2013; Drenzyk *et al*., 2014; Slesnick *et al*., 2015). Although many studies have focused on the search for self-efficacy factors that influence the behavior of patients with CKD and their implementation for patients with ESRD (Ferris *et al*., 2011; Flesher *et al*., 2011; Wierdsma *et al*., 2011; Enworom and Tabi, 2015; Montoya *et al*., 2016), studies describing the relationship between self-efficacy and self-management behavior can be used. In particular, the early stages of CKD are few, and their consequences are not clear. Information on such topics can be useful for the proper selection of relevant and sufficiently proven components of self-efficacy, which can lead to the development of a self-efficacy tool and self-management or intervention suitable for patients with early CKD.

Recommendations should include a greater emphasis on behavioral and socio-economic aspects in patients with hypertension with CKD in primary care. Self-care as a component of personal care cannot be simply effective in improving QOL of people with hypertension and CKD and social determinants. Along with this, cultural interventions aimed at improving the psychological state of women and housewives compared with men and increasing the social activity of older people and the retired should be noted. Although measuring QOL using tools such as QOL questionnaires is inevitable in quantitative research, different aspects of life for everyone have a different effect on the perception of their QOL. We recommend predicting models and elements when using such tools to calculate the final QOL score for the correct weighing of various aspects of QOL by any individual participant, then the final score will be a more accurate assessment of the QOL of this person.

It is also recommended that further intervention studies be done and actions such as self-care and self-management and participatory decision-making be compared and the best intervention be determined.

A logical first step to enhance the ability of hypertensive individuals with CKD to participate in recommended health behaviors is to address the barriers to behavior engagement. The more readily modifiable barriers include physical functioning, self-efficacy, social support, health literacy, and depressive symptoms, which could serve as potential targets for intervention. Addressing health literacy could be particularly beneficial as it has been associated with better outcomes in other chronic diseases, can lead to greater self-efficacy, which in turn leads to improved self-management, which includes participation in recommended health behaviors (Buchbinder *et al*., 2011). In addition, future directions in CKD management could further explore the role of self-efficacy in the patient−provider relationship, as well as conducting randomized clinical trials of health behavior promotion programs, exploring the role of supporting health behaviors to address poor outcomes.

Therefore, health-promoting behaviors in hypertensive patients with CKD have a potential impact on their QOL in primary care. Assessing QOL of patients with hypertension and CKD and paying attention to health-promoting behaviors are required for improving QOL. Attention to the QOL of hypertensive patients with CKD provides an overview of the patient’s health status.

## Limitations

There are several limitations to this study. First, the sample size was relatively small. In addition, the study was conducted at a single center. The study was an open-label design, and none of the participants were blinded to treatment assignment. Second, the instrument used to measure self-efficacy exhibited a low level of reliability, with a Cronbach’s *α* of 0.64, even though it did have an acceptable level of reliability in other studies.

## Conclusion

The findings of this study indicate that self-care health behaviors play an important role in QOL in patients with hypertension and concomitant CKD in primary care. The cardiovascular risk factors can be more effectively managed in the individuals performing the self-care health-promoting behaviors. Primary care physicians should focus on motivation strategies to encourage individuals to perform self-care health-promoting behaviors associated with the improved QOL, in order to achieve better outcomes in risk factor management.

## Data Availability

No additional data are available.
